# Review: The Potential of the Common Bean (*Phaseolus vulgaris*) as a Vehicle for Iron Biofortification

**DOI:** 10.3390/nu7021144

**Published:** 2015-02-11

**Authors:** Nicolai Petry, Erick Boy, James P. Wirth, Richard F. Hurrell

**Affiliations:** 1 Groundwork LLC, Crans-près-Céligny 1299 Switzerland; E-Mail: james@groundworkhealth.org; 2 International Food Policy Research Institute, Washington, DC 20006-1002, USA; E-Mail: E.Boy@cgiar.org; 3 Institute of Food, Nutrition and Health, Laboratory of Human Nutrition, ETH Zurich, 8092 Zurich, Switzerland; E-Mail: richard.hurrell@hest.ethz.ch

**Keywords:** common bean, iron biofortification, phytic acid, polyphenols, ferritin, stable iron isotope studies

## Abstract

Common beans are a staple food and the major source of iron for populations in Eastern Africa and Latin America. Bean iron concentration is high and can be further increased by biofortification. A major constraint to bean iron biofortification is low iron absorption, attributed to inhibitory compounds such as phytic acid (PA) and polyphenol(s) (PP). We have evaluated the usefulness of the common bean as a vehicle for iron biofortification. High iron concentrations and wide genetic variability have enabled plant breeders to develop high iron bean varieties (up to 10 mg/100 g). PA concentrations in beans are high and tend to increase with iron biofortification. Short-term human isotope studies indicate that iron absorption from beans is low, PA is the major inhibitor, and bean PP play a minor role. Multiple composite meal studies indicate that decreasing the PA level in the biofortified varieties substantially increases iron absorption. Fractional iron absorption from composite meals was 4%–7% in iron deficient women; thus the consumption of 100 g biofortified beans/day would provide about 30%–50% of their daily iron requirement. Beans are a good vehicle for iron biofortification, and regular high consumption would be expected to help combat iron deficiency (ID).

## 1. Introduction

The domestication of *Phaseolus vulgaris* (common bean) occurred independently in South America and Central America/Mexico, leading to two different domesticated gene pools, the Andean and Mesoamerican, respectively [1]. The common bean is currently estimated to be one of the most important legumes worldwide [2], and is an important source of nutrients for more than 300 million people in parts of Eastern Africa and Latin America, representing 65% of total protein consumed, 32% of energy [3,4,5], and a major source of micronutrients e.g., iron, zinc, thiamin and folic acid; [4,6,7]. The annual global bean production is approximately 12 million metric tons, with 5.5 and 2.5 million metric tons alone in Latin America Caribbean (LAC) and Africa, respectively [4,8]. The highest producer is India at more than 4 million metric tons per year [9]. LAC and African countries with the highest production are Brazil, Mexico, the Democratic Republic of the Congo (DRC), Kenya, Tanzania and Uganda. The highest apparent per capita consumption is found in Burundi, Kenya and Rwanda [3], ranging from 31 kg to 66 kg per year [4,8], equivalent to 180 g per capita and day. However, bean consumption and production tend to be underestimated because beans are often intercropped and consumed in remote rural areas [10] where dietary intake data are often incomplete or inexistent [11]. Thus, estimations of bean consumption as high as 200 g and 300 g per capita per day have been reported in Rwanda and certain regions of the DRC, respectively [12,13].

Although the average iron concentration in beans is high 55 μg/g; [14] compared to other major crops such as wheat [15], rice [16] and maize [17], many people living in these countries still suffer from ID due to an insufficient level of bioavailable iron in a monotonous cereal/bean-based diet without meat [18,19,20]. One potentially sustainable strategy to combat ID in bean-eating populations is iron biofortification. Beans exhibit sufficient genetic variability in iron concentration, which is the basic requirement for biofortification. The multidisciplinary biofortification approach could therefore be used to counteract ID by either increasing the concentration and/or bioavailability of iron in beans through traditional plant breeding, or by employing genetic engineering techniques [21].

In order to successfully introduce a biofortified crop in to the food system, other human and environmental factors have to be properly addressed. Although no behavioral changes are required from the consumers for invisible traits such as mineral biofortification, the target varieties have to be chosen carefully, following the consumer’s dietary patterns and culinary preferences [22]. Sensory and cooking qualities have to be maintained and studies assessing consumer preferences must be undertaken in different cultural settings [11]. The new variety also has to be accepted and cultivated by the farmers, and must exhibit high agronomic yield and resistance to pathogens and other environmental stresses; in short, it must be as or more profitable than local varieties. To augment the sustainability of biofortification in general, and beans in particular, breeders have to take into account the impact of climate, soils and agronomic practices on iron concentration [23,24].

In some countries (e.g., Rwanda and DRC), plant breeders have already developed and released new *P. vulgaris* bean varieties with iron concentrations above 94 μg/g, the target level of HarvestPlus, an international research program supporting the research and development of biofortified crops [25,26,27]. They show good micronutrient retention after processing, and equal or increased agronomic yield, indicating that the common bean may be a promising crop for iron biofortification [28]. However, successful bean iron biofortification might be constrained due to the reported low iron bioavailability associated with high concentrations of PA [29,30] and PP [14,31], two potentially potent iron absorption inhibitors in common beans [32,33,34,35,36]. Recent human stable isotope iron absorption studies, conducted with black and brown biofortified bean varieties, [37,38] reported that the additional iron bred into biofortified beans was of low bioavailability and the authors questioned whether biofortified beans could make a useful contribution to filling the gap between current iron intake and requirements [28].

This review evaluates the potential of the common bean as a vehicle for iron biofortification, with a focus on human studies of iron absorption from beans and bean-containing meals and the impact of compounds present in beans (PA; PP; proteins) on bean iron absorption.

## 2. Methods

Due to the broad scope of the review, key words related to the overarching topics were searched in PubMed and Web of Science to identify published literature related to bean consumption, bean production and iron in beans including iron biofortification, iron speciation, iron absorption inhibitors and human studies conducted with beans and bean containing meals. The key-word search was conducted in September 2014, and included the following words and expressions: (common bean * OR Phaseolus vulgaris *) and (iron * OR iron biofortification * OR consumption * OR production * OR iron absorption * OR iron bioavailability * OR isotope studies * OR iron absorption inhibitors * OR phytate * OR polyphenols * OR proteins * OR lectin * OR ferritin *). Additional sources (published and unpublished) were identified through a reference review of key publications and theses [39,40,41] and following discussions with researchers at HarvestPlus and ETH Zurich.

Sources not pertaining to the aforementioned topics (e.g., bean consumption, production, biofortification, iron speciation and iron absorption) were not included in this review; in total, 212 published and unpublished sources were included in this review.

## 3. Results and Discussion

### 3.1. Iron in Beans

#### 3.1.1. Genetic Variability of Iron Concentrations in Beans

Iron in beans is present in higher concentrations than in cereal staples, and is almost completely retained through harvest and processing [14,42]. More than 36,000 accessions of beans for 44 species of *Phaseolus* from 109 countries are held in a gene bank at the “Centro Internacional de Agricultura Tropical” (CIAT) in Cali, Colombia, making it the most diverse and largest bean collection worldwide [43]. Much data are available on the iron content of beans, but the most complete overview and reliable information is provided by two independently conducted studies screening the common bean core collection of CIAT, which is a systematic sample of the germplasm available and contains more than 1000 genotypes. Both studies reported that there is a promising genetic variability for iron in beans [14,44] with iron concentrations ranging from about 35–90 μg/g, with an average of 55 μg/g. Iron concentration in 119 wild varieties tested was only slightly higher than in the cultivated beans with an average Fe concentration of 60 μg/g [14]. Other researchers reported much higher iron concentrations in wild types ranging from 71 μg/g to 280 μg/g [45], and suggested that these wild varieties be used in biofortification programs to increase iron concentration in cultivated varieties. It is not clear to what extent these very high iron concentrations are due to iron contamination from soil or other sources, but this is a potential source of apparent variability that should be investigated.

There is no correlation between geographic distribution and iron concentration in beans, although beans from the Andean gene pool tend to have higher iron concentrations than Mesoamerican beans [14]. However, variability of iron concentration in beans was not only ascribed to bean variety, but was also influenced by the planting site and season [46].

#### 3.1.2. Iron Speciation in Beans

**Figure 1:**
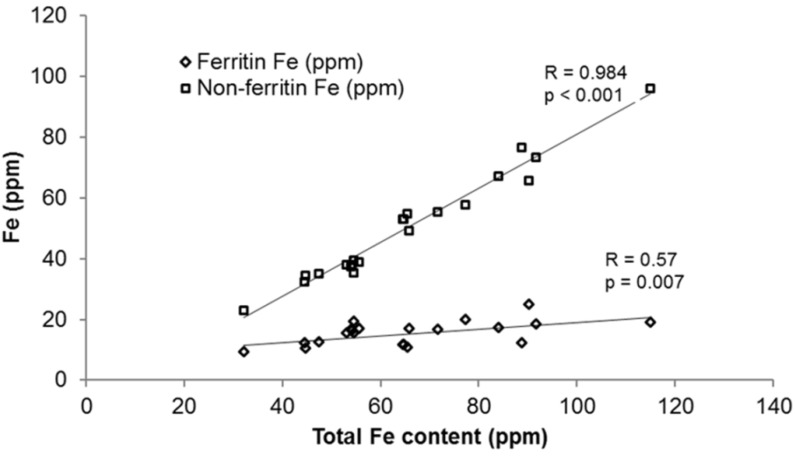
Scatterplot (including regression lines) showing the correlation between total iron content of 21 common bean genotypes *versus* their ferritin-bound iron (◊) and non-ferritin-bound iron (□) fractions [53].

Storage iron in legumes is sequestered in ferritin, which is the major iron storage protein. Ferritin consists of 24 protein subunits that can store up to 4500 Fe^3+^ atoms in the form of an iron oxyhydroxide-phosphate mineral [47,48]. It is abundant in legumes and has been reported in beans, soybeans, lentils and peas [49,50,51,52]. Hoppler and colleagues [52] recently developed an isotope dilution method to quantify ferritin in different legume seeds and reported that the concentration of ferritin-bound iron in beans was lower than previously reported using other techniques [48] and ranged from 15% to 30% of total iron. Thus, 70%–85% of the iron present in beans is in the form of non-ferritin-bound iron possibly bound to PA. Hoppler and colleagues [53], using their isotope dilution technique [52] subsequently reported that ferritin concentration in beans is independent of iron concentration and that as the iron concentration in beans increases, there is an increase in the non-ferritin bound iron (Figure 1). They further observed a correlation between non-ferritin bound iron and phytate and suggested that this might be the reason for the low iron bioavailability reported from biofortified beans. In colored beans, it is also possible that iron in the seed coat [54] is bound to PP as little PA is located in the seed coat.

Although early studies reported poor iron absorption from animal ferritin [55,56,57,58,59], this seems to be due to an inappropriate labeling procedure [60], and recent data indicate that plant ferritin-iron is as well absorbed by humans as ferrous sulfate and the common inorganic iron pool [61,62]. There has been speculation that ferritin iron is absorbed intact by a separate mechanism and that the iron is protected from PA. This led to the proposal that increasing ferritin iron in plant foods by plant breeding programs could be a solution to global ID. This hypothesis assumes that much of the ferritin survives cooking and digestion, protects iron from absorption inhibitors such as PA and calcium in the gastrointestinal tract, and is absorbed intact (with its high iron concentration) via a ferritin specific transporter [63]. This proposal is based on a series of human and Caco-2 cell studies made by Theil and colleagues [63,64,65,66]. They showed in the human iron bioavailability studies that ferritin iron absorption was not influenced by certain concentrations of hemoglobin and ferrous sulfate and they suggested that ferritin iron is absorbed by a different mechanism than iron salts/chelates or heme iron and that ferritin was unaffected by gastric and luminal digestion [63].

This suggestion, however, is not supported by radioisotope studies in humans with intrinsically labeled black beans that reported equal absorption from the intrinsic and extrinsic radio labels, indicating that all the iron species in the bean were absorbed to the same extent as common pool iron [67], and by *in vitro* studies which reported that ferritin iron is readily and completely released from the ferritin molecule during cooking and gastric digestion [68,69,70]. These studies suggest that the absorption of ferritin iron in beans would be equally influenced by inhibitors and enhancers of iron absorption as non-ferritin iron. Further studies may be needed to resolve these contradictory results.

One way forward would be the measurement of iron absorption in human subjects from intrinsically labeled beans that contain different proportions of total iron as ferritin iron. However, at present the weight of the evidence suggests that iron speciation in beans is relatively unimportant as iron appears to be readily and completely released from ferritin by cooking and digestion [69] and, in the same way as non-ferritin iron, ferritin iron after its release would be expected to bind to PA in the gastric juice forming insoluble, non-bioavailable complexes [37,71].

#### 3.1.3. Progress in Bean Iron Biofortification

The initial goal of the HarvestPlus bean biofortification initiative was to use selective plant breeding strategies to produce bean varieties with at least 80% more iron than found in conventional beans [28]. The targeted iron level for beans was 94 μg/g, which represented an increase of 44 μg/g as compared to the average concentration in the germplasm. Assuming a mean iron absorption of 5%, the target increase was estimated to meet one third of the daily iron needs of the most vulnerable population groups who consumed 30%–40% of their daily calories from beans [13]. The target level was quickly reached [17,26,37,72], and the first human studies testing the performance of biofortified beans have already been conducted [37,73].

Several approaches were used to breed high iron beans. Blair and colleagues [25] developed a high iron bean line by an advanced backcross breeding approach including backcrossing, recurrent selection and various permutations of gamete and pedigree selection. The new bean line, which was derived by backcrossing a high iron wild type bean into a commonly cultivated bean from the Andean gene pool, had an iron concentration ranging from 92 μg/g to 99 μg/g [25]. Using a different approach, the same researchers [26] developed two promising new, red mottled Andean bush beans with improved iron and zinc concentrations. The lines were derived by crossing a red mottled bean, commonly cultivated in eastern and southern Africa, and in the Andean region, with a brown seeded high mineral climbing bean. The agronomic performance of the new varieties was tested in the Andean region and Central America. Iron and zinc concentrations strongly depended on the planting site but were on average 18–23 mg/kg higher than in the red mottled parental bean. Although affected by environmental factors, the higher iron concentration in the biofortified beans compared to the parent beans over different environments indicates that breeding for high iron should be successful. However, although high iron genotypes will accumulate more iron than the low iron genotypes grown at the same location during the same growing season [14,46], the major challenge will be to maintain high iron concentration in a sufficient number of genotypes to cover varying climates, altitudes and soil types. In Rwanda progress has been made to address this challenge, with ten high-iron varieties released since 2010 that cover different growth types (bush and climbers) and agro-ecological zones (low, mid and high altitude), and span a wide range of market classes (seed color, grain size, cooking time).

Another approach to boost the iron concentration in beans focuses on interspecific crosses with *P dumosus* and *P coccineus*. Results of interspecific crosses are promising especially for Mesoamerican beans where it has proven difficult to get iron levels above 90 ppm [74]. A further step forward, which offers new insights into inheritance of bean iron concentration, is the recent identification of the quantitative gene loci (QTL) that control iron accumulation [42,46]. These findings are promising for the use in biofortification because they give more precise genetic information about targeted traits, rendering marker-assisted selection of high micronutrient beans possible.

An alternative to plant breeding is agronomic biofortification with the application of mineral fertilizers to soils or leaves. Agronomic biofortification through soil fertilization has increased zinc [75,76] and selenium levels [77] in cereals but has been much less successful for iron [78]. The usefulness of iron containing fertilizers added to soils is hindered by the rapid and strong binding of the added iron to soil particles which prevents its uptake by the plant [79]. Better results might be achieved with the application of chelates such as FeEDTA because more iron remains bioavailable in solution [80,81]. But these fertilizers are more expensive [81] and bear the risk of leaching because they increase mineral mobility throughout the whole soil [82,83]. Another method of agronomic biofortification is foliar application of zinc fertilizers, which is more effective than zinc soil application and has been shown to increase the Zn grain yield of wheat and rice [84,85,86]. In contrast, the application of iron fertilizers has only little or no effect on grain iron [87,88], but increasing the supply of N boosts both iron and zinc concentration in the grain and shoot [87]. Fertilizers, however, are costly and they must be applied repeatedly, which might raise the question of compatibility between the application of fertilizers and the philosophy of sustainable biofortification.

### 3.2. Compounds Influencing Iron Absorption from Beans

#### 3.2.1. Polyphenols-Impact on Iron Absorption and Human Health

PP are a heterogeneous class of compounds derived from the secondary plant metabolism. They protect the plant against pathogens and UV radiation, and play an important role in pollination by insects [89,90,91]. Their ability to form non-absorbable complexes with iron in the intestinal tract, as well as the strength and the nature of bonding, depends to a large extent on the polyphenol’s structure [36,92]. PP with only one donor atom available to bind to the iron atom, form rather weak complexes with the iron, whereas bidentate PP, which bind iron through two sites can be very powerful ligands [93]. It is suggested that for the PP to effectively bind the iron, at least two hydroxyl groups in the *ortho*- position are necessary [94], and if the hydroxyl groups are arranged differently, the PP behaves like a monodendate ligand [93,95].

The inhibitory nature of PP present in different plant foods has been the subject of many human isotopic absorption studies, and PP from vegetables, legumes, spices, red wine, different teas, cocoa and coffee decrease iron absorption [36,96,97,98,99,100]. The strong inhibitory effect of sorghum PP on iron absorption has been observed in several absorption studies [34,101] and recently published data suggest that 162 mg sorghum PP added to a non-inhibitory phytate-free bread meal decrease iron absorption by about 70% [102]. This compares to a reduction of almost 80% by the same concentration black tea PP added to a similar bread meal [36].

While it is tempting to suggest decreasing PP in beans as a means to improve iron nutrition, the reported health benefits of certain PP should be considered. Some monomeric PP can be absorbed and are reported to have physiological effects leading to health benefits. However, they need to be present at a sufficiently high concentration and be adequately absorbed if they are to exert biological effects [103]. Polymeric PP are not absorbed but are extensively degraded by fermentation in the colon to a variety of metabolites that are absorbed and may also have beneficial physiological effects [104]. There is epidemiologic evidence that certain PP reduce the risk of several forms of cancers [105], and that individuals with high flavonoid intake have a reduced risk of cardiovascular disease [106,107,108,109,110,111]. The European Food Safety Agency has recently accepted a health claim that cocoa flavanols improve blood flow [112]. The mechanism of action is thought to be via an influence of monomeric epicatechin on the enzymes producing nitric oxide, which leads to a relaxation of the blood vessels [113,114] and potentially a decrease in blood pressure. Although PP compounds *in vitro* are strong antioxidants [115], they are extensively modified on absorption [116] and can lose much of their antioxidant potential. Nevertheless, it is possible that they could help prevent oxidative stress by trapping OH radicals [117,118] or by forming complexes with iron and preventing its participation in the Fenton reaction [119,120], which leads to free radical production and possible tissue damage.

#### 3.2.2. Occurrence of Different PP in Common Beans

**Figure 2:**
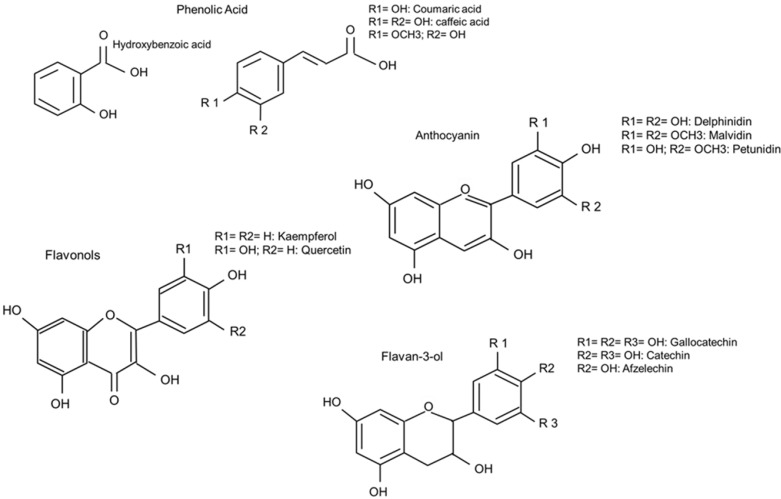
The predominant PP units in beans that occur mostly in various polymeric forms.

Common beans contain a wide range of PP including phenolic acids, flavanols (flavan-3-ols; e.g., catechin, gallocatechin, afzelechin), anthocyanidins (e.g., delphinidin, cyanidin, mainly present in glycosylated form) as well as flavonols (e.g., quercetin, kaempferol; Figure 2), the latter three classes being responsible for bean pigmentation [121].

Flavanols mostly occur in the form of oligomers and polymers, which are called proanthocyanidins or condensed tannins [122]. However, PP content and profile differ widely between beans and are determined by the bean variety and seed color [14]. In addition, analytical values for total PP concentration differ strongly depending on the chemical assay used.

As quantified by the simpler, colorimetric Folin Ciocalteau method, white beans have by far the lowest total PP concentration, ranging from 40 to 80 mg PP/100 g beans and red colored beans tend to have the highest PP concentrations with up to 800 mg/100 g expressedingallicacidequivalents; [123]. The major PP in colored beans are the proanthocyanidins, which are primarily located in the bean hull [29,124]. Diaz and colleagues [125] found concentrations ranging from 10% to 30% of seed coat weight, with an overall average of about 20% in 250 bean varieties. Since bean hulls account for approximately 7% of total bean weight, the amount found in hulls was equivalent to total proanthocyanidin concentrations of up to 2 g/100 g bean. Lower proanthocyanidin concentrations ranging from 0.2 to 1.1 g/100 g bean [31,126] have also been reported. Using mass spectrometry, the majority of bean proanthocyanidins are reported to be procyanidins that are made up of catechin and epicatechin units; [31,127]. Prodelphinidins (polymers of (epi)-gallocatechin), have also been reported in beans by Aparicio-Fernandez and colleagues [127], but were not found by Gu *et al.* [128].

Flavonol glycosides, such as quercetin and kaempferol, are exclusively present in colored beans [31,129,130]. However, flavonol, as well as proanthocyanidin concentration in beans, is influenced by the length of seed storage [31]. Intensely colored beans are rich in anthocyanins, the glycosylated anthocyanidins, which are also exclusively located in the seed coat. Black beans were found to contain up to 218 mg/100 g anthocyanins, with highest concentrations in delphinidin 3- glycoside (56%), but no anthocyanins were found in white beans [131,132]. Wu and colleagues identified delphinidin-, malvidin- and petunidin glycosides in black beans, whereas red beans contained cyanidin- and pelargonidin glycosides [133]. Beans also contain numerous phenolic acids, including hydroxybenzoic, p-coumaric, caffeic and ferulic acids [134], but at very low concentrations. Based on the literature, it would appear that most of the PP present in colored beans are polymeric. The levels of potentially absorbable monomeric flavanols and phenolic acids are low. The polymeric PP are largely based on flavanols that have at least two hydroxyl groups in the *ortho*- position and possess the potential to complex iron and perhaps other minerals. It is also apparent that PP concentrations and the PP profile in beans vary widely depending on genotype and color. Data suggest that PP levels in colored beans can vary more widely within a single color class than between the different color classes. This would allow selecting for low PP traits in the different bean color classes [14]. To our knowledge, there are no specific reports of the beneficial effects of PP from beans on human health.

#### 3.2.3. Phytic Acid-Impact on Iron Absorption and Human Health

*Myo*-inositol-1,2,3,4,5,6-hexakisphosphate (PA) is the most abundant phosphorylated *myo*- inositol derivative. Phytate, the salt of PA, is ubiquitous in eukaryotic species and serves as the major phosphorous and mineral storage form. It accounts for 1%–5% of the composition of legumes, cereals, oil seeds, pollens, and nuts. Phytate is mainly located in the kernel, where it contains up to 75% of the plant’s phosphorous, whereas roots and other plant compartments contain smaller quantities [135,136]. In most cereals, phytate is located in the aleurone layer, pericarp and the germ [137], whereas in legumes the highest concentrations can be found in the protein bodies of the endosperm or the cotyledon [138].

PA is highly negatively charged under physiological conditions and therefore forms strong, highly insoluble complexes with divalent and monovalent minerals such as iron, zinc, magnesium, copper, calcium and potassium, thus providing the plant with essential minerals for normal ripening and maturation [139], signaling [140] and responding to plant pathogens [141]. It is estimated that the daily consumption in industrialized countries ranges from 0.3 to 2.6 g per day, whereas PA intake is much higher in developing countries, where people mainly consume diets based on plants. Detailed information about food sources, intake, processing and bioavailability is available through a recently published review [138].

PA has a strong negative effect on iron absorption [32] and can decrease iron status [142]. Several isotope absorption studies in humans have also shown PA to inhibit zinc [143], calcium [144], magnesium [145] and manganese [146] absorption. PA’s particularly strong inhibition of iron absorption was shown in single meal isotope absorption studies [32], in which little up-regulation of iron absorption during long-term PA consumption occurred [147]. However, interpretation of these results may be complicated by the fact that single meal studies tend to overestimate the effect of inhibitors on iron absorption and an adaptation of the human organism to inhibitors might take place over the long-term [148]. This is supported by a recently developed algorithm on multiple meal studies indicating that iron status is a more important predictor of iron absorption than dietary factors [149].

The effect of PA on iron absorption is dose dependent. Hallberg and colleagues [33] showed that 10 mg/100 g, 20 mg/100 g and 100 mg/100 g PA reduced iron absorption by 20%, 40% and 60%, respectively in a bread meal free of iron absorption enhancers. This dose dependency was confirmed in another study, which, showed that the inhibition of PA on iron absorption can be counteracted by the addition of ascorbic acid [33,150]. EDTA is a fortification compound that also has the ability to increase iron absorption from meals rich in PA [151] and iron EDTA is the fortification compound recommended for high phytate food vehicles [152]. With cereal- and legume-based foods, the enzymatic degradation of phytate using added exogenous phytases, either during processing [153] or immediately prior to meal consumption [151], or by activating native phytases during processing [154] can substantially improve iron bioavailability. It has been suggested that, in terms of the inhibition of iron absorption, the PA: iron molar ratio is more important than the total amount of PA [155]. With simple meals devoid of absorption enhancers, it has been recommended that PA: iron molar ratio should be below 1:1 and preferably 0.4:1 [153]. In composite meals with meat or vegetables containing ascorbic acid or other enhancers, a PA: iron molar ratio <6:1 is proposed [155,156].

In addition to degrading PA concentrations enzymatically, PA can be decreased by mechanical removal [157] or by plant breeding [158]. The mechanical removal of PA is not easily applicable to beans because the phytate is mainly in the cotyledon [30]. The activity of native, endogenous phytases in cereals and legumes was investigated by Egli and colleagues [159]. They reported phytase activity in beans to be relatively low compared to wheat, barley and rye, but in the same range as in other legumes. Germination, but not soaking, increased bean phytase activity, as was reported for other legumes such as mung beans [160]. Endogenous phytases can be activated by reducing pH with fermentation, and encouraging results have been reported with pearl millet [161]. This approach is less applicable to beans that are usually consumed whole and not fermented.

While it is clear that a decrease in PA would in many cases benefit iron nutrition, PA like PP has been proposed to have potential health benefits and a decrease in PA is not exclusively perceived as positive. The ability of PA to bind iron and prevent possible free radical generation has led to the speculation that PA may have health-promoting properties particularly in the prevention of certain cancers [138,162].

As PA is not absorbed in humans, it has been most investigated in relation to colon cancer where unabsorbed iron may be a risk factor through the generation of free radical [163,164], which could react with the colonic mucosa. It has been suggested that PA exerts a positive impact in the colon by binding free unabsorbed iron, and thus preventing the formation of iron generated free radicals, and also by the direct up-regulation of tumor suppressor genes [162,165,166]. However, most information on PA as a therapeutic compound has been derived from cell studies and observational human studies [167] and, although the potential for PA to bind iron in the colon exists, iron is mostly insoluble at colonic pH [168,169].

#### 3.2.4. Phytic Acid in Common Beans

Most of the PA present in beans (95%–98%) is located in the cotyledon, whereas the embryo (1%–3%) and the seed coat (0.5%–4%) contain small quantities [30]. A wide variation in PA concentration, ranging from 400 to 2600 mg/100 g beans has been reported [29,30,45,170], with a mean content of about 1000 mg/100 g beans and a PA:iron molar ratio ranging from 6:1 to 33:1. The PA level depends on the bean variety, environment and the analytical assay used for PA quantification. An impact of growing site and soil type on the total PA concentration in beans has been reported by Blair and colleagues [170]. They observed significant differences in PA in the same bean varieties grown on high or medium phosphorous soil [158].

There is also evidence that the bean PA concentration is influenced by the bean iron concentration. Results from Hoppler *et al.* [53] showed that PA simultaneously increases with the iron concentration in beans (Figure 3), although there is a slight decrease in the PA: iron molar ratio. This is in accordance with the results of other studies, where beans also had the tendency to accumulate more PA with higher iron concentration [45].

**Figure 3:**
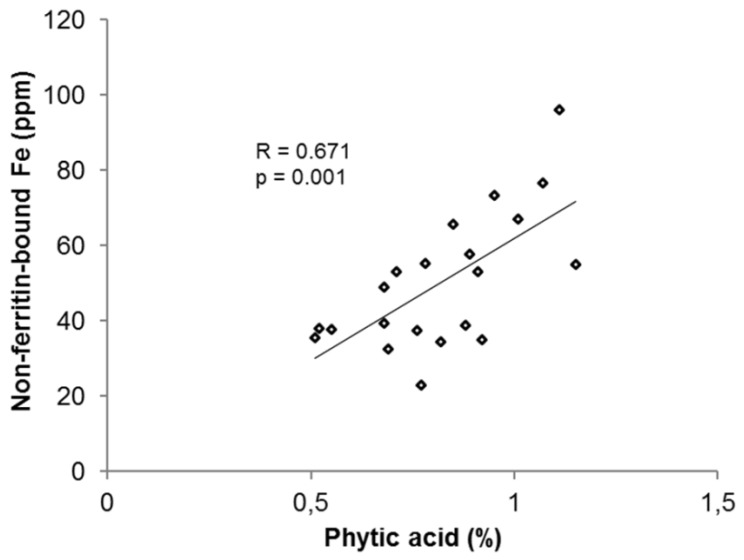
3. Correlation between non-ferritin-bound iron in ppm and PA in g/100 g bean [53].

The hypothesis that PA concentration is correlated with iron concentration is further strengthened by recent studies testing the performance of iron-biofortified beans in humans [37,38]. Both iron biofortified beans investigated had much higher PA concentration than the control beans indicating that additional iron bred into the beans was associated with additional PA accumulation. However, breeding simultaneously for high iron and low PA should be possible since, as mentioned above, Blair and colleagues have reported that most phytate and phosphorous related QTL are independent of iron QTL [158]. There are also data from studies with beans [171], cowpeas [172] and wheat [173] that indicate that the PA concentration is not associated with yield or plant health.

Campion and colleagues have recently used mutagenesis to develop a low phytic acid (lpa) bean with normal phosphorus, but only 10% of the original PA concentration [171]. The mutation was associated with a defective gene, coding for an ATP-binding cassette (ABC) transporter taking part in PA storage in protein bodies during seed maturation [174]. Despite the large reduction in PA, this mutation retained a good grain yield and a high germination rate [171], in contrast to many previously developed lpa crops [175]. Free or weakly bound iron was increased seven-fold in the lpa beans [171] and a stable iron isotope absorption study in humans found a markedly improved iron bioavailability when compared to the parent beans [176]. The reduction of PA, by disrupting its biosynthetic chain, might therefore be a possible solution to alleviate the nutritional issues associated with high PA concentrations.

#### 3.2.5. Proteins-Impact on Iron Absorption

Food proteins can both enhance or inhibit iron absorption, either forming soluble complexes with iron and improving iron uptake, or forming insoluble unabsorbable complexes preventing iron uptake. The most prominent and widely examined proteins affecting iron bioavailability are the muscle tissue commonly referred to as the “meat factor”. Many studies have shown that the addition of muscle tissue to meals significantly improves non-heme iron absorption [177,178,179]. Identification of the “meat factor”, however, has proven to be difficult and it may not be a single compound. In early studies, the amino acid cysteine was shown to stimulate iron absorption in humans, and it was hypothesized that low molecular weight peptides containing cysteine might be part of the meat factor [180,181,182]. Adding the cysteine containing peptide glutathione [180], or an amino acid mixture as present in fish [183] to black bean meals strongly increased iron bioavailability in humans.

More recent studies by Storcksdieck *et al.* proposed that the “meat effect” was due to the rapid digestion of actin and myosin in the stomach and the ability of the many small peptides entering the duodenum to keep iron in solution and available for absorption [184]. Most other proteins are little digested in the stomach. Different types of glycosaminoglycans (GAG), which are part of the connective tissue between muscle fibers, have also been the subject of investigations. However, although GAGs promoted iron uptake in Caco-2 cells [185,186], a human study failed to show an effect on iron absorption [187]. Armah and colleagues [188] have also suggested that phosphatidyl choline contributes to the enhancing effect of meat. They showed an enhancing effect in a human study, which however could not be confirmed by others [151].

Other proteins of animal origin affecting iron absorption are found in milk and eggs [189]. Several researchers reported an inhibitory effect of milk proteins [189] and egg white/albumin [189,190], although the latter to a lesser extent [191]. Hurrell *et al.* conducted a human absorption study and showed that casein and whey proteins had a stronger negative impact on iron bioavailability than egg white [191], and other studies reported that replacing egg albumin or casein with soy products significantly reduced iron absorption in humans [192].

Studies with soy and other legume proteins are probably more relevant to the potential influence of bean proteins on iron absorption. Studies with soy are complicated by the high level of PA, but one of the major soy proteins in the globulin fraction, conglycinin, has shown to be inhibitory in the absence of PA [32,193]. However, not all legume proteins are inhibitory. The much higher iron bioavailability from a dephytinized pea protein isolate [194] compared to soy protein isolates [195] was explained by the absence of the conglycinin fraction in peas [153,194]. Finally from rat studies, there is evidence that isolated soybean lectin and concanavalin A might inhibit iron absorption [196].

#### 3.2.6. Proteins in Common Beans

Beans contain between 20% and 30% proteins. Although they are rich in some essential amino acids, such as leucine and lysine [197,198], the protein quality is limited by its low digestibility [199] and the low concentration of tryptophan and of sulfur containing amino acids [197,198]. These amino acids occur at low levels in the major seed storage protein phaseolin, which accounts for 30%–50% of total protein. Phaseolin belongs to the globulin family, which at 65% of the total protein is the most prevalent group of proteins in beans [199] and other legumes such as peas and soybeans [194]. It is present in beans in different forms, depending on its polypeptide composition [199]. It is not known whether bean phaseolin has a similar inhibitory effect on iron absorption in humans as the conglycinin fraction of soy bean protein; however, evidence from *in vitro* studies indicates that it can form insoluble complexes with iron [200,201].

Carrasco-Castilla and colleagues [200] tested the iron binding capacity of different phaseolin peptide fractions and enzyme hydrolysates of bean protein. Iron chelating activity was measured spectrometrically with ferrozine. The highest iron binding capacity (>80%) was observed with the phaseolin hydrolysate, although the total bean protein isolate after hydrolysis itself bound up to 36% of the iron. In another *in vitro* study, the same researchers investigated the iron chelating activity of total bean protein, phaseolin and extracted lectin. The unhydrolyzed lectin extract and phaseolin complexed 32% and 18% of the iron, respectively, but the unhydrolysed bean protein isolate had no effect on iron. Enzyme hydrolysis of total protein and phaseolin resulted in a strong increase in their iron binding activity, whereas lectin was less active after hydrolysis [201]. These results indicate that phaseolin in beans may negatively influence iron absorption in humans but that lectins are unlikely to have an effect, especially in cooked beans because they are heat labile compounds. This, however, remains to be tested.

### 3.3. Bean Iron Bioavailability

Almost all information about bean iron bioavailability in humans is from iron isotope studies using radio-or stable isotope extrinsic tagging techniques, which are commonly used tools to measure iron bioavailability from foods. Small quantities of iron isotopes are added as an extrinsic tag to one food in one or several test meals and iron absorption is measured as iron isotope incorporation into hemoglobin in the erythrocytes [148,202]. Although the validity of the method has been questioned under certain conditions in a review by Consaul and Lee [203], several human studies have proven this method to deliver reliable results by comparing extrinsic with intrinsic tagging in beans and other foods [67,73,204,205]. The majority of studies conducted with beans have been single/double meal studies. Compared to multiple meal studies such a study design might overestimate the effect of inhibitors and enhancers on mineral absorption [206,207], and results are more susceptible to intra-subject day-to-day variation in iron absorption. This has to be taken into consideration when interpreting the studies described below that investigated iron absorption from beans and bean-containing meals.

All studies have reported relatively low iron bioavailability from beans with absorption varying from below 1% to about 9%, depending on the bean/meal composition, study design, and the iron status of the study subjects. Cook and colleagues [67] first reported a very low (1.5%) mean fractional absorption in a radio-iron isotope study in 8 healthy subjects (4 male; 4 female) who consumed a simple meal containing mashed black beans. Similarly low iron bioavailability (<1%) from black beans was reported in a subsequent radio iron study conducted in 10 iron replete male subjects who consumed a soup containing beans, bay leaves, garlic powder, onions and red pepper [208]. In addition to the inhibitory effect of PA and PP, which were not quantified, another major reason for the low iron bioavailability in these studies was the relatively good iron status of the subjects.

#### 3.3.1. The Impact of Bean PP on Human Iron Absorption

Petry and colleagues investigated the impact of bean PP and PA on iron absorption from beans in a series of single meal stable isotope studies in humans [35]. In order to measure the impact of bean PP on iron absorption in the absence of PA, increasing amounts of red bean hulls were added as the source of bean PP to non-inhibitory bread meals in which PA had been completely degraded during fermentation of the dough. Twenty mg of bean PP (as measured by Folin-Ciocalteu) had no impact on iron absorption, whereas 50 mg and 200 mg reduced iron bioavailability by 18% and 45%, respectively, indicating that red bean PP caused a dose-dependent inhibition of iron bioavailability. Compared to PP from different beverages tested with a similar bread meal, PP from beans were less inhibitory than PP in black tea and a series of herb teas [36]. A second study also looked at the inhibition of bean PP in the absence of PA. In this study, homogenized dephytinized beans were fed with or without the PP-containing hulls. Removing the hulls doubled iron absorption from 3.5% to 7%.

Beans, however, are never consumed in the absence of PA, and in the presence of PA, bean PP appear to add little or no further inhibition to iron absorption above that caused by PA. In a single meal study conducted in Rwandan women with low iron status, iron absorption from a white bean meal consisting of 75 g (dry weight) pureed beans with a PP concentration of 65 mg was compared to iron absorption from a 75 g (dry weight) pureed red bean meal with a PP concentration of 260 mg. Both meals had comparable PA and iron concentrations. Iron absorption from the low PP bean meal (4.7%) was 27% higher than from the high PP meal (*p* < 0.05). However, the effect of PP was no longer observed when the beans were fed with potatoes or rice in a multiple meal design [37]. Iron absorption values from both meals containing either the high PP or the low PP beans, were about 7%. Other studies have also failed to show an impact of bean PP on iron absorption in the presence of PA [176,209]. Beiseigel and colleagues [209] for example, compared iron absorption from the low PP Great Northern bean (white/crème) to iron absorption from the high PP Pinto bean (marbled brown) in a single meal study with healthy women with normal iron status. Beans had comparable iron and PA concentrations. Iron absorption from both beans was about 2%.

There is one published iron absorption study with beans that still needs an explanation. It was part of the series of studies made by Petry *et al.* [35]. In this study, beans with their natural PA concentration were fed with their PP-containing hull intact or with the hull removed. Iron absorption significantly decreased with the removal of most of the PP’s with the hull. The authors speculated that this was due to an enhancing interaction between a carbohydrate component of the hull and PA, rather than due to the removal of PP, which were unlikely to have enhanced iron absorption.

In summary, several single meal iron absorption studies in humans clearly show that PP of colored beans can have a negative impact on iron absorption in the absence of high PA concentrations, and that the extent of the inhibition depends on the PP profile and level. Bean PP, however, are less inhibitory than PP from teas and in the presence of PA, add little or no further inhibition. Additionally, no negative effect of bean PP has been observed in multiple meal studies. It is unlikely therefore that bean PP will influence iron status in the real life situation when beans are eaten as part of the composite daily diet and where the impact of inhibitors and enhancers is more equilibrated.

#### 3.3.2. The Impact of Bean-PA on Human Iron Absorption

PA, on the other hand, is a more potent inhibitor of bean iron absorption, and its impact has been shown in single [35,73,176] as well as multiple meal studies [37,38]. Studies conducted with biofortified beans have given new insights on the impact of PA on bean iron absorption, indicating perhaps that the total amount of PA or the additional PA present in biofortified beans, and not the PA:iron molar ratio, determines iron bioavailability [37,73]. Petry and colleagues carried out a multiple meal study in Rwanda, a bean-consuming population. They compared iron absorption from a biofortified high iron bean (92 μg iron/g bean; 13,900 μg PA/g bean) administered over several days as part of composite meals to iron absorption from a normal iron bean (52 μg iron/g bean; 8500 μg PA/g bean) [37].

Beans had similar PA:iron molar ratios (9:1). Relative iron bioavailability from the biofortified high iron bean was significantly lower (3.8%) than from the normal iron bean (6.3%), leading to equal amounts of total iron absorbed from the two tested beans. The authors suggested that the observed effect was due to the higher concentration of PA in the biofortified bean.

**Figure 4:**
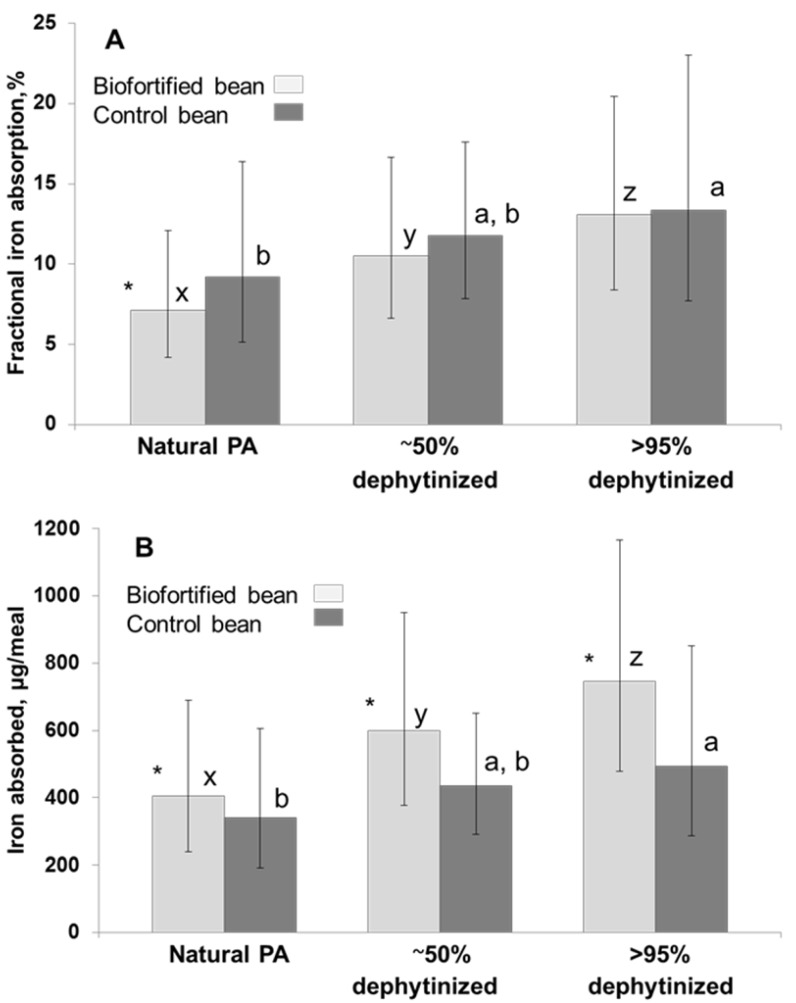
Fractional iron absorption and total amount of iron absorbed from a biofortified and a control bean with natural PA level, after partial and almost total dephytinization [38].

To further test this hypothesis, another multiple composite meal study with biofortified and control carioca beans of the same color was conducted in the same Rwandan study population [38]. The iron absorption from the biofortified (88 μg iron/g) and the control bean (54 μg iron/g) was compared. Beans were served either with their natural PA concentration (biofortified bean: 12000 μg PA/g bean; control bean: 9000 μg PA/g bean), partially (about 50%) dephytinized (biofortified bean: 5500 μg PA/g bean; control bean: 3610 μg PA/g bean) or almost totally dephytinized (about 5% PA; biofortified bean: 450 μg PA/g bean; control bean: 450 μg PA/g bean). Again, fractional iron absorption from the biofortified bean (7.1%) was significantly lower (*p* < 0.05) than from the control bean (9.2%), when beans had their natural PA concentration. Partial dephytinization increased bioavailable iron mainly in the biofortified bean (+48%), which led to equal fractional iron absorption and to an increase of more than 35% in the total amount of iron absorbed from the biofortified bean. This effect was even more pronounced after the removal of 95% of PA and participants absorbed about 50% more iron from the biofortified bean compared to the control bean (Figure 4).

Petry and colleagues recently conducted a paired double-meal crossover study with lpa bean seeds with both high and low PP and their parent beans with normal PA levels [176]. Lpa seeds were derived by chemical mutagenesis and had only a small fraction of their original PA concentration (5%–10%). Highest iron absorption of 6.1% was observed from the lpa seed high in PP (PP, 140 mg/meal; PA, 25 mg/meal), followed by 4% iron absorption from the lpa seed low in PP (PP, 32 mg/meal; PA, 45 mg/meal). Iron absorption from the parent beans high in PA (590 and 690 mg/meal) was 3.8% and 2.7% respectively. This was unexpected as iron absorption from beans in the absence of PA has been reported to depend on PP concentration [35]. The authors suggested that their findings were due to the much stronger inhibitory nature of PA compared to the PP present in the beans. The PA concentration in the lpa bean high in PP (PA: iron molar ratio 0.6:1) was about half that of the lpa bean low in PP (PA: iron molar ratio 1.2:1), resulting in a higher absorption even in the presence of the higher PP content. This study confirmed that PA is the major inhibitor of iron absorption from beans and indicates that the lpa mutation could be introduced in beans independent of the PP concentration and thus independent of bean color.

### 3.4. Can Iron-Biofortified Beans be an Efficacious Intervention to Combat Iron Deficiency?

In bean-consuming communities, the high iron level in the biofortified varieties coupled with the relatively high bean consumption and modest iron absorption indicate that beans should be a good vehicle for iron biofortification, despite their high PA content. Although iron absorption from beans is relatively low, biofortified beans have a much higher iron level than regular beans and with a daily consumption of 100 g biofortified beans provide about 10 mg iron, a 5 mg increase in daily iron intake as compared to consuming the same quantity of regular beans. The observed fractional iron absorption from composite bean meals of around 4%–7% would thus provide an adult women with 400–700 μg absorbed iron per 100 g biofortified beans, an increase as compared to the regular beans. Consumption of 100 g biofortified beans in a composite meal with potato or rice could therefore provide up to half of the daily absorbable iron requirement of women of child bearing age [210].

Experience from efficacy studies that have demonstrated improved iron status in populations consuming iron-fortified foods can be used to evaluate the potential of iron-biofortified staples. Hallberg *et al.* [211] estimated that it took 2–3 years to stabilize a new iron balance when increasing the bioavailable iron intake, although 80% of the final impact is achieved in the first year, and around 40% of final impact in 5–6 months. Most iron fortification efficacy studies that have demonstrated improved iron status, have provided the iron-fortified foods for 6–9 months. The successful ferrous sulfate efficacy studies in women or children of marginal iron status indicated that an additional 7 mg additional iron per day was necessary to significantly improve iron status [152].

Showing efficacy with iron-biofortified beans will be more difficult than for most iron-fortified foods as the additional daily iron provided by biofortified beans is restricted at present to about 5 mg/100 g, and the iron bioavailability will be less due to the high PA and lack of ascorbic acid or EDTA recommended for high phytate fortified foods [19]. More time might be needed to demonstrate improved iron status in efficacy studies with biofortified beans. The first studies with biofortified beans have been completed in Mexico and Rwanda (although not yet published), indicating that biofortified beans can be efficacious at improving iron status [212].

## 4. Conclusions

Beans are a good source of iron compared to other staples and a promising vehicle for iron biofortification. Although they are high in PA, bioavailability studies have demonstrated that the proposed targeted iron bioavailability of 5% [11] can be achieved when beans are consumed as part of composite meals in a multiple meal design administered to women of low iron status. Multiple meal studies are the most appropriate tool to determine iron bioavailability from whole diets, and can be taken to indicate long-term potential. Great progress has been made by exploiting the genetic iron variability of beans and target iron levels have been reached and even exceeded. On the other hand, due to the low bioavailability of bean iron shown in isotope studies, exclusively breeding for high iron concentration may not provide enough additional absorbable iron to impact iron status. PA has been demonstrated to be the major determinant of iron bioavailability from beans and, to optimize iron absorption from biofortified beans, the focus must now be on PA reduction. There is little information to suggest a target PA level for iron-biofortified beans, which would depend to some extent on the meal composition. Our suggestion is 7000 μg PA/g iron-biofortified bean, as recent studies indicate that PA concentrations <7000 μg PA/g bean would usefully improve iron absorption from multiple composite meals compared to varieties containing >12,000 μg PA/g bean. If the increase in absorbed iron is still not sufficient to fill the gap between absorbed iron and iron requirement, lpa bean lines could be explored as a possible solution.

## References

[1] Debouck D.G., Toro O., Paredes O.M., Johnson W.C., Gepts P. (1993). Genetic diversity and ecological distribution of *Phaseolus vulgaris* (Fabaceae) in northwestern South America. Econ. Bot..

[2] HarvestPlus Iron-bean, 2009. http://www.harvestplus.org/sites/default/files/HarvstPlus_Bean_Strategy.pdf.

[3] Blair M., Gonzales L.F., Kimani P.M., Butare L. (2010). Genetic diversity, inter-gene pool introgression and nutritional quality of common beans (*Phaseolus vulgaris* L.) from central africa. Theor. Appl. Genet..

[4] Broughton W.J., Hernandez G., Blair M., Beebe S., Gepts P., Vanderleyden J. (2003). Beans (*Phaseolus* spp.)-model food legumes. Plant Soil.

[5] Welch R.M., House W.A., Beebe S., Cheng Z. (2000). Genetic selection for enhanced bioavailable levels of iron in bean (*Phaseolus vulgaris* L.) seeds. J. Agric. Food Chem..

[6] Pennington J.A.T., Young B. (1990). Iron zinc copper manganese selenium and iodine in foods from the United States total diet study. Food Compost. Anal..

[7] Souci S.W., Fachmann W., Kraut H. (1994). Food Composition and Nutrition Tables.

[8] CGIAR Common Bean, 2012. http://www.cgiar.org/our-research/crop-factsheets/beans/.

[9] FAO Food Balance Sheet, 2012. http://faostat3.fao.org/faostat-gateway/go/to/download/FB/FBS/E.

[10] Jones A.L. Phaseolus Bean: Post-Harvest Operations, 1999. http://www.fao.org/fileadmin/user_upload/inpho/docs/Post_Harvest_Compendium_-_Phaesolus_beans.pdf.

[11] Hotz C., McClafferty B. (2007). From harvest to health: Challenges for developing biofortified staple foods and determining their impact on micronutrient status. Food Nutr. Bull..

[12] HarvestPlus Rwanda, 2009. http://www.harvestplus.org/content/iron-beans-rwanda.

[13] HarvestPlus DRC, 2009. http://www.harvestplus.org/content/iron-beans-dr-congo.

[14] Beebe S., Gonzalez A.V., Rengifo J. (2000). Research on trace minerals in the common bean. Food Nutr. Bull..

[15] Ortiz-Monasterio J.I., Palacios-Rojas N., Meng E., Pixley K., Trethowan R., Pena R.J. (2007). Enhancing the mineral and vitamin content of wheat and maize through plant breeding. J. Cereal. Sci..

[16] Gregorio G.B. (2002). Progress in breeding for trace minerals in staple crops. J. Nutr..

[17] Bänziger M., Long J. (2000). The potential for increasing the iron and zinc density through plant-breeding. Food Nutr. Bull..

[18] Bouis H.E. (2007). The potential of genetically modified food crops to improve human nutrition in developing countries. J. Dev. Stud..

[19] WHO (2006). Guidelines on Food Fortification with Micronutrients.

[20] Baynes R.D., Bothwell T.H. (1990). Iron-deficiency. Annu. Rev. Nutr..

[21] White P.J., Broadley M.R. (2005). Biofortifying crops with essential mineral elements. Trends Plant. Sci..

[22] Qaim M., Stein A.J., Meenakshi J.V. (2007). Economics of biofortification. Agric. Econ. Blackwell.

[23] Gilligan D.O. (2012). Biofortification, agricultural technology adoption, and nutrition policy: Some lessons and emerging challenges. CESifo Econ. Stud..

[24] Oury F.X., Leenhardt F., Remesy C., Chanliaud E., Duperrier B., Balfourier F., Charmet G. (2004). Genetic variability and stability of grain magnesium, zinc and iron concentrations in bread wheat. Proceedings of the International Workshop on Modelling Quality Traits and Their Genetic Variability for Wheat.

[25] Blair M.W., Izquierdo P. (2012). Use of the advanced backcross-QTL method to transfer seed mineral accumulation nutrition traits from wild to Andean cultivated common beans. Theor. Appl. Genet..

[26] Blair M.W., Monserrate F., Beebe S.E., Restrepo J., Flores J.O. (2010). Registration of high mineral common bean germplasm lines NUA35 and NUA56 from the red-mottled seed class. J. Plant. Regist..

[27] HarvestPlus Crop Development And Delivery Schedule Iron Bean DRC, 2014. http://www.harvestplus.org/sites/default/files/Delivery%20Schedule_Iron%20Bean%20DR%20Congo.pdf.

[28] Bouis H.E., Welch R.M. (2010). Biofortification—A sustainable agricultural strategy for reducing micronutrient malnutrition in the global south. Crop Sci..

[29] Anton A., Ross K., Beta T., Fulcher R., Arntfield S. (2008). Effect of pre-dehulling treatments on some nutritional and physical properties of navy and pinto beans (*Phaseolus vulgaris* L.). LWT-Food Sci. Technol..

[30] Ariza-Nieto M., Blair M.W., Welch R.M., Glahn R.P. (2007). Screening of iron bioavailability patterns in eight bean (*Phaseolus vulgaris* L.) genotypes using the caco-2 cell *in vitro* model. J. Agric. Food Chem..

[31] Beninger C.W., Gu L.W., Prior R.L., Junk D.C., Vandenberg A., Bett K.E. (2005). Changes in polyphenols of the seed coat during the after-darkening process in pinto beans (*Phaseolus vulgaris* L.). J. Agric. Food Chem..

[32] Hurrell R.F., Juillerat M.A., Reddy M.B., Lynch S.R., Dassenko S.A., Cook J.D. (1992). Soy protein, phytate, and iron-absorption in humans. Am. J. Clin. Nutr..

[33] Hallberg L., Brune M., Rossander L. (1989). Iron-absorption in man-ascorbic-acid and dose-dependent inhibition by phytate. Am. J. Clin. Nutr..

[34] Hurrell R.F., Reddy M.B., Juillerat M.A., Cook J.D. (2003). Degradation of phytic acid in cereal porridges improves iron absorption by human subjects. Am. J. Clin. Nutr..

[35] Petry N., Egli I., Zeder C., Walczyk T., Hurrell R. (2010). Polyphenols and phytic acid contribute to the low iron bioavailability from common beans in young women. J. Nutr..

[36] Hurrell R.F., Reddy M., Cook J.D. (1999). Inhibition of non-haem iron absorption in man by polyphenolic-containing beverages. Br. J. Nutr..

[37] Petry N., Egli I., Gahutu J.B., Tugirimana P.L., Boy E., Hurrell R. (2012). Stable iron isotope studies in Rwandese women indicate that the common bean has limited potential as a vehicle for iron biofortification. J. Nutr..

[38] Petry N., Egli I., Gahutu J.B., Tugirimana P.L., Boy E., Hurrell R. (2014). Phytic acid concentration influences iron bioavailability from biofortified beans in Rwandese women with low iron status. J. Nutr..

[39] Bonsmann S.S.G. (2006). Dietary Factors Infleuncing Non-Heme Iron Absorption. Ph.D. Thesis.

[40] Hoppler M. (2008). Content and Bioavailability of Ferritin-Bound Iron in Staple Food Crops. Ph.D. Thesis.

[41] Petry N. (2011). Biofortification: Optimizing Iron Absorption from Beans and Other Staple Foods. Ph.D. Thesis.

[42] Blair M.W., Medina J.I., Astudillo C., Rengifo J., Beebe S.E., Machado G., Graham R. (2010). QTL for seed iron and zinc concentration and content in a mesoamerican common bean (*Phaseolus vulgaris* L.) population. Theor. Appl. Genet..

[43] CIAT *Genetic* Resources Program-Bean Collection, 2011; International Center for Tropical Agriculture. http://isa.ciat.cgiar.org/urg/beancollection.do;jsessionid=3DD436BFD78E7AB8BC00871432B44819.

[44] Islam F.M.A., Basford K.E., Jara C., Redden R.J., Beebe S. (2002). Seed compositional and disease resistance differences among gene pools in cultivated common bean. Genet. Resour. Crop Evol..

[45] Guzman-Maldonado S.H., Acosta-Gallegos J., Paredes-Lopez O. (2000). Protein and mineral content of a novel collection of wild and weedy common bean (*Phaseolus vulgaris* L.). J. Sci. Food Agric..

[46] Blair M., Astudillo C., Grusak M., Graham R., Beebe S. (2009). Inheritance of seed iron and zinc concentrations in common bean (*Phaseolus vulgaris* L.). Mol. Breed..

[47] Harrison P.M., Arosio P. (1996). Ferritins: Molecular properties, iron storage function and cellular regulation. Bba Bioenerg..

[48] Theil E.C. (2004). Iron, ferritin, and nutrition. Annu. Rev. Nutr..

[49] Laulhere J.P., Lescure A.M., Briat J.F. (1988). Purification and characterization of ferritins from maize, pea, and soybean seeds-distribution in various pea organs. J. Biol. Chem..

[50] Sczekan S.R., Joshi J.G. (1987). Isolation and characterization of ferritin from soybeans (glycine-max). J. Biol. Chem..

[51] Wardrop A.J., Wicks R.E., Entsch B. (1999). Occurrence and expression of members of the ferritin gene family in cowpeas. Biochem. J..

[52] Hoppler M., Zeder C., Walczyk T. (2009). Quantification of ferritin-bound iron in plant samples by isotope tagging and species-specific isotope dilution mass spectrometry. Anal. Chem..

[53] Hoppler M., Egli I., Petry N., Gille D., Zeder C., Walczyk T., Blair M.W., Hurrell R.F. (2014). Iron speciation in beans (*Phaseolus vulgaris*) biofortified by common breeding. J. Food Sci..

[54] Blair M.W., Izquierdo P., Astudillo C., Grusak M.A. (2013). A legume biofortification quandary: Variability and genetic control of seed coat micronutrient accumulation in common beans. Front. Plant Sci..

[55] Derman D.P., Bothwell T.H., Torrance J.D., Macphail A.P., Bezwoda W.R., Charlton R.W., Mayet F.G.H. (1982). Iron-absorption from ferritin and ferric hydroxide. Scand. J. Haematol..

[56] Hussain R., Walker R.B., Layrisse M., Clark P., Finch C.A. (1965). Nutritive value of food iron. Am. J. Clin. Nutr..

[57] Layrisse M., Martineztorres C., Renzy M., Leets I. (1975). Ferritin iron-absorption in man. Blood.

[58] Martinez-Torres C., Leets I., Taylor P., Ramirez J., Camacho M.D., Layrisse M. (1986). Heme, ferritin and vegetable iron-absorption in humans from meals denatured of heme iron during the cooking of beef. J. Nutr..

[59] Skikne B., Fonzo D., Lynch S.R., Cook J.D. (1997). Bovine ferritin iron bioavailability in man. Eur. J. Clin. Nutr..

[60] Beard J.L., Burton J.W., Theil E.C. (1996). Purified ferritin and soybean meal can be sources of iron for treating iron deficiency in rats. J. Nutr..

[61] Lonnerdal B., Bryant A., Liu X.F., Theil E.C. (2006). Iron absorption from soybean ferritin in nonanemic women. Am. J. Clin. Nutr..

[62] Davila-Hicks P., Theil E.C., Lonnerdal B. (2004). Iron in ferritin or in salts (ferrous sulfate) is equally bioavailable in nonanemic women. Am. J. Clin. Nutr..

[63] Theil E.C., Chen H.J., Miranda C., Janser H., Elsenhans B., Nunez M.T., Pizarro F., Schumann K. (2012). Absorption of iron from ferritin is independent of heme iron and ferrous salts in women and rat intestinal segments. J. Nutr..

[64] Kalgaonkar S., Lonnerdal B. (2008). Effects of dietary factors on iron uptake from ferritin by caco-2 cells. J. Nutr. Biochem..

[65] Kalgaonkar S., Lonnerdal B. (2009). Receptor-mediated uptake of ferritin-bound iron by human intestinal caco-2 cells. J. Nutr. Biochem..

[66] Martin C.D.S., Garri C., Pizarro F., Walter T., Theil E.C., Nunez M.T. (2008). Caco-2 intestinal epithelial cells absorb soybean ferritin by mu2 (AP2)-dependent endocytosis. J. Nutr..

[67] Cook J.D., Finch C.A., Walker R., Martinez C., Layrisse M., Monsen E. (1972). Food iron absorption measured by an extrinsic tag. Clin. Investig..

[68] Bejjani S., Pullakhandam R., Punjal R., Nair K.M. (2007). Gastric digestion of pea ferritin and modulation of its iron bioavailability by ascorbic and phytic acids in caco-2 cells. World J. Gastroenterol..

[69] Hoppler M., Schonbachler A., Meile L., Hurrell R.F., Walczyk T. (2008). Ferritin-iron is released during boiling and *in vitro* gastric digestion. J. Nutr..

[70] Jin F.X., Frohman C., Thannhauser T.W., Welch R.M., Glahn R.P. (2009). Effects of ascorbic acid, phytic acid and tannic acid on iron bioavailability from reconstituted ferritin measured by an *in vitro* digestion-caco-2 cell model. Br. J. Nutr..

[71] Makower R.U. (1970). Extraction and determination of phytic acid in beans (*Phaseolus vulgaris*). Cereal Chem..

[72] Ribeiro N.D., Domingues L.D., Zemolin A.E.M., Possobom M.T.D. (2013). Selection of common bean lines with high agronomic performance and high calcium and iron concentrations. Pesqui. Agropecu. Bras..

[73] Donangelo C.M., Woodhouse L.R., King S.M., Toffolo G., Shames D.M., Viteri F.E., Cheng Z., Welch R.M., King J.C. (2003). Iron and zinc absorption from two bean (*Phaseolus vulgaris* L.) genotypes in young women. J. Agric. Food Chem..

[74] Blair M., Astudillo C., Beebe S., Roa I., Kimani P.M., Chirwa R.M. Biofortification Breeding of Common Bean (*Phaseolus vulgaris* L.), 2009. http://www.biokemi.org/biozoom/issues/525/articles/2397.

[75] Shehu H.E., Jamala G.Y. (2010). Available Zn distribution, response and uptake of rice (*Oriza sativa*) to applied zn along a topose quence of lake gerio fadama soils at Yola, North-eastern Nigeria. J. Am. Sci..

[76] Cakmak I. (2008). Enrichment of cereal grains with zinc: Agronomic or genetic biofortification?. Plant Soil.

[77] Hawkesford M.J., Zhao F.J. (2007). Strategies for increasing the selenium content of wheat. J. Cereal Sci..

[78] Cakmak I., Pfeiffer W.H., McClafferty B. (2010). Biofortification of durum wheat with zinc and iron. Cereal Chem..

[79] Hirschi K.D. (2009). Nutrient biofortification of food crops. Annu. Rev. Nutr..

[80] Rengel Z., Batten G.D., Crowley D.E. (1999). Agronomic approaches for improving the micronutrient density in edible portions of field crops. Field Crop Res..

[81] Khoshgoftarmanesh A.H., Schulin R., Chaney R.L., Daneshbakhsh B., Afyuni M. (2010). Micronutrient-efficient genotypes for crop yield and nutritional quality in sustainable agriculture. A review. Agron. Sustain. Dev..

[82] Alvarez J.M., Novillo J., Obrador A., Lopez-Valdivia L.M. (2001). Mobility and leachability of zinc in two soils treated with six organic zinc complexes. J. Agric. Food Chem..

[83] Gonzalez D., Obrador A., Alvarez J.M. (2007). Behavior of zinc from six organic fertilizers applied to a navy bean crop grown in a calcareous soil. J. Agric. Food Chem..

[84] Zou C.Q., Zhang Y.Q., Rashid A., Ram H., Savasli E., Arisoy R.Z., Ortiz-Monasterio I., Simunji S., Wang Z.H., Sohu V. (2012). Biofortification of wheat with zinc through zinc fertilization in seven countries. Plant Soil.

[85] Cakmak I., Kalayci M., Kaya Y., Torun A.A., Aydin N., Wang Y., Arisoy Z., Erdem H., Yazici A., Gokmen O. (2010). Biofortification and localization of zinc in wheat grain. J. Agric. Food Chem..

[86] Boonchuay P., Cakmak I., Rerkasem B., Prom-U-Thai C. (2013). Effect of different foliar zinc application at different growth stages on seed zinc concentration and its impact on seedling vigor in rice. Soil Sci. Plant Nutr..

[87] Aciksoz S.B., Yazici A., Ozturk L., Cakmak I. (2011). Biofortification of wheat with iron through soil and foliar application of nitrogen and iron fertilizers. Plant Soil.

[88] Zhang Y.Q., Shi R.L., Rezaul K.M., Zhang F.S., Zou C.Q. (2010). Iron and zinc concentrations in grain and flour of winter wheat as affected by foliar application. J. Agric. Food Chem..

[89] Manach C., Scalbert A., Morand C., Remesy C., Jimenez L. (2004). Polyphenols: Food sources and bioavailability. Am. J. Clin. Nutr..

[90] Friedman M. (1997). Chemistry, biochemistry, and dietary role of potato polyphenols. A review. J. Agric. Food Chem..

[91] Sisa M., Bonnet S.L., Ferreira D., van der Westhuizen J.H. (2010). Photochemistry of flavonoids. Molecules.

[92] Brune M., Rossander L., Hallberg L. (1989). Iron-absorption and phenolic-compounds-importance of different phenolic structures. Eur. J. Clin. Nutr..

[93] Hider R.C., Liu Z.D., Khodr H.H. (2001). Metal chelation of polyphenols. Method Enzymol..

[94] Brune M., Hallberg L., Skanberg A.B. (1991). Determination of iron-binding phenolic groups in foods. J. Food Sci..

[95] Purawatt S., Siripinyanond A., Shiowatana J. (2007). Flow field-flow fractionation-inductively coupled optical emission spectrometric investigation of the size-based distribution of iron complexed to phytic and tannic acids in a food suspension: Implications for iron availability. Anal. Bioanal. Chem..

[96] Hallberg L., Rossander L. (1982). Absorption of iron from western-type lunch and dinner meals. Am. J. Clin. Nutr..

[97] Disler P.B., Lynch S.R., Charlton R.W., Torrance J.D., Bothwell T.H., Walker R.B., Mayet F. (1975). Effect of tea on iron-absorption. Gut.

[98] Cook J.D., Reddy M.B., Hurrell R.F. (1995). The effect of red and white wines on nonheme-iron absorption in humans. Am. J. Clin. Nutr..

[99] Gillooly M., Bothwell T.H., Torrance J.D., Macphail A.P., Derman D.P., Bezwoda W.R., Mills W., Charlton R.W., Mayet F. (1983). The effects of organic-acids, phytates and polyphenols on the absorption of iron from vegetables. Br. J. Nutr..

[100] Tuntipopipat S., Judprasong K., Zeder C., Wasantwisut E., Winichagoon P., Charoenkiatkul S., Hurrell R., Walczyk T. (2006). Chili, but not turmeric, inhibits iron absorption in young women from an iron-fortified composite meal. J. Nutr..

[101] Gillooly M., Bothwell T.H., Charlton R.W., Torrance J.D., Bezwoda W.R., Macphail A.P., Derman D.P., Novelli L., Morrall P., Mayet F. (1984). Factors affecting the absorption of iron from cereals. Br. J. Nutr..

[102] Cercamondi C.I., Egli I.M., Zeder C., Hurrell R.F. (2014). Sodium iron edta and ascorbic acid, but not polyphenol oxidase treatment, counteract the strong inhibitory effect of polyphenols from brown sorghum on the absorption of fortification iron in young women. Br. J. Nutr..

[103] Manach C., Williamson G., Morand C., Scalbert A., Remesy C. (2005). Bioavailability and bioefficacy of polyphenols in humans. I. Review of 97 bioavailability studies. Am. J. Clin. Nutr..

[104] Selma M.V., Espin J.C., Tomas-Barberan F.A. (2009). Interaction between phenolics and gut microbiota: Role in human health. J. Agric. Food Chem..

[105] Dai J., Mumper R.J. (2010). Plant phenolics: Extraction, analysis and their antioxidant and anticancer properties. Molecules.

[106] Taubert D., Berkels R., Roesen R., Klaus W. (2003). Chocolate and blood pressure in elderly individuals with isolated systolic hypertension. JAMA J. Am. Med. Assoc..

[107] Rein D., Paglieroni T.G., Wun T., Pearson D.A., Schmitz H.H., Gosselin R., Keen C.L. (2000). Cocoa inhibits platelet activation and function. Am. J. Clin. Nutr..

[108] Heiss C., Dejam A., Kleinbongard P., Schewe T., Sies H., Kelm M. (2003). Vascular effects of cocoa rich in flavan-3-ols. JAMA J. Am. Med. Assoc..

[109] Grassi D., Desideri G., Croce G., Tiberti S., Aggio A., Ferri C. (2009). Flavonoids, vascular function and cardiovascular protection. Curr. Pharm. Design..

[110] Sesso H.D., Gaziano J.M., Buring J.E., Hennekens C.H. (1999). Coffee and tea intake and the risk of myocardial infarction. Am. J. Epidemiol..

[111] Mukamal K.J., Maclure M., Muller J.E., Sherwood J.B., Mittleman M.A. (2002). Tea consumption and myocardial mortality after acute infarction. Circulation.

[112] EFSA Scientific opinion on the substantiation of a health claim related to cocoa flavanols and maintenance of normal endothelium-dependent vasodilation pursuant to Article 13(5) of Regulation (EC) No 1924/2006. European Food and Safelty Authority. http://www.efsa.europa.eu/en/search/doc/2809.pdf.

[113] Njike V.Y., Faridi Z., Shuval K., Dutta S., Kay C.D., West S.G., Kris-Etherton P.M., Katz D.L. (2011). Effects of sugar-sweetened and sugar-free cocoa on endothelial function in overweight adults. Int. J. Cardiol..

[114] Sies H., Schewe T., Heiss C., Kelm M. (2005). Cocoa polyphenols and inflammatory mediators. Am. J. Clin. Nutr..

[115] Ghasemzadeh A., Ghasemzadeh N. (2011). Flavonoids and phenolic acids: Role and biochemical activity in plants and human. J. Med. Plants Res..

[116] Lee H.C., Jenner A.M., Low C.S., Lee Y.K. (2006). Effect of tea phenolics and their aromatic fecal bacterial metabolites on intestinal microbiota. Res. Microbiol..

[117] Van Acker S.A.B.E., van Balen G.P., van den Berg D.J., Bast A., van der Vijgh W.J.F. (1998). Influence of iron chelation on the antioxidant activity of flavonoids. Biochem. Pharmacol..

[118] Perron N.R., Brumaghim J.L. (2009). A review of the antioxidant mechanisms of polyphenol compounds related to iron binding. Cell Biochem. Biophys..

[119] Yoshino M., Murakami K. (1998). Interaction of iron with polyphenolic compounds: Application to antioxidant characterization. Anal. Biochem..

[120] Chaudhary P., Shukla S.K., Kumar I.P., Namita I., Afrin F., Sharma R.K. (2006). Radioprotective properties of apple polyphenols: An *in vitro* study. Mol. Cell. Biochem..

[121] Caldas G.V., Blair M.W. (2009). Inheritance of seed condensed tannins and their relationship with seed-coat color and pattern genes in common bean (*Phaseolus vulgaris* L.). Theor. Appl. Genet..

[122] Escarpa A., Gonzalez M.C. (2001). An overview of analytical chemistry of phenolic compounds in foods. Crit. Rev. Anal. Chem..

[123] Tajeri Foman J. (2006). Evaluation potentieller Strategien zur Eisenbiofortifizierung von *Phaseolus vulgaris*.

[124] Reddy N.R., Pierson M.D., Sathe S.K., Salunkhe D.K. (1985). Dry bean tannins: A review of nutritional implications. J. Am. Oil Chem. Soc..

[125] Diaz A.M., Caldas G.V., Blair M.W. (2010). Concentrations of condensed tannins and anthocyanins in common bean seed coats. Food Res. Int..

[126] Maldonado S.H.G., MarinJarillo A., Castellanos J.Z., DeMejia E.G., AcostaGallegosc J.A. (1996). Relationship between physical and chemical characteristics and susceptibility to *Zabrotes subfasciatus* (Boh.) (Coleoptera:Bruchidae) and *Acanthoscelides obtectus* (Say) in common bean (*Phaseolus vulgaris* L.) varieties. J. Stored Prod. Res..

[127] Aparicio-Fernandez X., Yousef G.G., Loarca-Pina G., de Mejia E., Lila M.A. (2005). Characterization of polyphenolics in the seed coat of black jamapa bean (*Phaseolus vulgaris* L.). J. Agric. Food Chem..

[128] Gu L.W., Kelm M.A., Hammerstone J.F., Beecher G., Holden J., Haytowitz D., Gebhardt S., Prior R.L. (2004). Concentrations of proanthocyanidins in common foods and estimations of normal consumption. J. Nutr..

[129] Lin L., Harnly J., Pastor-Corrales M., Luthria D. (2008). The polyphenolic profiles of common bean (*Phaseolus vulgaris* L.). Food Chem..

[130] Espinosa-Alonso L.G., Lygin A., Widholm J.M., Valverde M.E., Paredes-Lopez O. (2006). Polyphenols in wild and weedy mexican common beans (*Phaseolus vulgaris* L.). J. Agric. Food Chem..

[131] Choung M.G., Choi B.R., An Y.N., Chu Y.H., Cho Y.S. (2003). Anthocyanin profile of Korean cultivated kidney bean (*Phaseolus vulgaris* L.). J. Agric. Food Chem..

[132] Takeoka G.R., Dao L.T., Full G.H., Wong R.Y., Harden L.A., Edwards R.H., Berrios J.D. (1997). Characterization of black bean (*Phaseolus vulgaris* L.) anthocyanins. J. Agric. Food Chem..

[133] Wu X.L., Prior R.L. (2005). Identification and characterization of anthocyanins by high-performance liquid chromatography-electrospray ionization-tandem mass spectrometry in common foods in the United States: Vegetables, nuts, and grains. J. Agric. Food Chem..

[134] Laparra J.M., Glahn R.P., Miller D.D. (2008). Bioaccessibility of phenols in common beans (*Phaseolus vulgaris* L.) and iron (Fe) availability to caco-2 cells. J. Agric. Food Chem..

[135] Raboy V. (2003). Myo-inositol-1,2,3,4,5,6-hexakisphosphate. Phytochemistry.

[136] Cheryan M. (1980). Phytic acid interactions in food systems. CRC Crit. Rev. Food Sci. Nutr..

[137] O’dell B.L., de Boland A.R., Koirtyohann S.R. (1972). Distribution of phytate and nutritionally important elements among morphological components of cereal grains. J. Agric. Food Chem..

[138] Schlemmer U., Frolich W., Prieto R.M., Grases F. (2009). Phytate in foods and significance for humans: Food sources, intake, processing, bioavailability, protective role and analysis. Mol. Nutr. Food Res..

[139] Reddy N.R., Sathe S.K. (2002). Food Phytates.

[140] Bohn L., Meyer A.S., Rasmussen S.K. (2008). Phytate: Impact on environment and human nutrition. A challenge for molecular breeding. J. Zhejiang Univ.Sci. B.

[141] Murphy A.M., Otto B., Brearley C.A., Carr J.P., Hanke D.E. (2008). A role for inositol hexakisphosphate in the maintenance of basal resistance to plant pathogens. Plant J..

[142] Zimmermann M.B., Chaouki N., Hurrell R.F. (2005). Iron deficiency due to consumption of a habitual diet low in bioavailable iron: A longitudinal cohort study in moroccan children. Am. J. Clin. Nutr..

[143] Navert B., Sandstrom B., Cederblad A. (1985). Reduction of the phytate content of bran by leavening in bread and its effect on zinc-absorption in man. Br. J. Nutr..

[144] Weaver C.M., Heaney R.P., Martin B.R., Fitzsimmons M.L. (1991). Human calcium-absorption from whole-wheat products. J. Nutr..

[145] Bohn T., Davidsson L., Walczyk T., Hurrell R.F. (2004). Phytic acid added to white-wheat bread inhibits fractional apparent magnesium absorption in humans. Am. J. Clin. Nutr..

[146] Davidsson L., Almgren A., Juillerat M.A., Hurrell R.F. (1995). Manganese absorption in humans: The effect of phytic acid and ascorbic acid in soy formula. Am. J. Clin. Nutr..

[147] Brune M., Rossander L., Hallberg L. (1989). Iron-absorption-no intestinal adaptation to a high-phytate diet. Am. J. Clin. Nutr..

[148] Hunt J.R., Roughead Z.K. (2000). Adaptation of iron absorption in men consuming diets with high or low iron bioavailability. Am. J. Clin. Nutr..

[149] Armah S.M., Carriquiry A., Sullivan D., Cook J.D., Reddy M.B. (2013). A complete diet-based algorithm for predicting nonheme iron absorption in adults. J. Nutr..

[150] Siegenberg D., Baynes R.D., Bothwell T.H., Macfarlane B.J., Lamparelli R.D., Car N.G., Macphail P., Schmidt U., Tal A., Mayet F. (1991). Ascorbic-acid prevents the dose-dependent inhibitory effects of polyphenols and phytates on nonheme-iron absorption. Am. J. Clin. Nutr..

[151] Troesch B., Egli I., Zeder C., Hurrell R.F., de Pee S., Zimmermann M.B. (2009). Optimization of a phytase-containing micronutrient powder with low amounts of highly bioavailable iron for in-home fortification of complementary foods. Am. J. Clin. Nutr..

[152] Hurrell R., Ranum P., de Pee S., Biebinger R., Hulthen L., Johnson Q., Lynch S. (2010). Revised recommendations for iron fortification of wheat flour and an evaluation of the expected impact of current national wheat flour fortification programs. Food Nutr. Bull..

[153] Hurrell R.F. (2004). Phytic acid degradation as a means of improving iron absorption. Int. J. Vitam. Nutr. Res..

[154] Sandberg A.S. (2002). Bioavailability of minerals in legumes. Br. J. Nutr..

[155] Hurrell R., Egli I. (2010). Iron bioavailability and dietary reference values. Am. J. Clin. Nutr..

[156] Tuntawiroon M., Sritongkul N., Rossanderhulten L., Pleehachinda R., Suwanik R., Brune M., Hallberg L. (1990). Rice and iron-absorption in man. Eur. J. Clin. Nutr..

[157] Hotz C., Gibson R.S. (2007). Traditional food-processing and preparation practices to enhance the bioavailability of micronutrients in plant-based diets. J. Nutr..

[158] Blair M.W., Herrera A.L., Sandoval T.A., Caldas G.V., Filleppi M., Sparvoli F. (2012). Inheritance of seed phytate and phosphorus levels in common bean (*Phaseolus vulgaris* L.) and association with newly-mapped candidate genes. Mol. Breed..

[159] Egli I., Davidsson L., Juillerat M.A., Barclay D., Hurrell R.F. (2002). The influence of soaking and germination on the phytase activity and phytic acid content of grains and seeds potentially useful for complementary feeding. J. Food Sci..

[160] Marero L.M., Payumo E.M., Aguinaldo A.R., Matsumoto I., Homma S. (1991). Antinutritional factors in weaning foods prepared from germinated cereals and legumes. Food Sci. Technol. Leb..

[161] Sharma A., Kapoor A.C. (1996). Levels of antinutritional factors in pearl millet as affected by processing treatments and various types of fermentation. Plant Food Hum. Nutr..

[162] Shamsuddin A.M. (2002). Anti-cancer function of phytic acid. Int. J. Food Sci. Tech..

[163] Kelly C. (2002). Can excess iron increase the risk for coronary heart disease and cancer?. Nutr. Bull..

[164] Radulescu S., Brookes M.J., Salgueiro P., Ridgway R.A., McGhee E., Anderson K., Ford S.J., Stones D.H., Iqbal T.H., Tselepis C. (2012). Luminal iron levels govern intestinal tumorigenesis after apc loss *in vivo*. Cell Rep..

[165] Coradini D., Pellizzaro C., Marimpietri D., Abolafio C., Daidone M.G. (2000). Sodium butyrate modulates cell cycle-related proteins in HT29 human colonic adenocarcinoma cells. Cell Proliferat..

[166] Saied H.T., Shamsuddin A.M. (1998). Up-regulation of the tumor suppressor gene p53 and WAF1 gene expression by IP6 in HT-29 human colon carcinoma cell line. Anticancer Res..

[167] Kumar V., Sinha A.K., Makkar H.P.S., Becker K. (2010). Dietary roles of phytate and phytase in human nutrition: A review. Food Chem..

[168] Ohkawara Y., Bamba M., Nakai I., Kinka S., Masuda M. (1963). Absorption of iron from human large intestine. Gastroenterology.

[169] Crichton R.R. (2001). Solution Chemistry of Iron in Biological Media. Inorganic Biochemistry of Iron Metabolism: From Molecular Mechanisms to Clinical Consequences.

[170] Barampama Z., Simard R.E. (1993). Nutrient composition, protein-quality and antinutritional factors of some varieties of dry beans (*Phaseolus vulgaris*) grown in Burundi. Food Chem..

[171] Campion B., Sparvoli F., Doria E., Tagliabue G., Galasso I., Fileppi M., Bollini R., Nielsen E. (2009). Isolation and characterisation of an *lpa* (low phytic acid) mutant in common bean (*Phaseolus vulgaris* L.). Theor. Appl. Genet..

[172] Afiukwa C.A., Igwenyi I.O., Ogah O., Offor C.E., Ugwu O.O. (2011). Variations in seed phytic and oxalic acid contents among nigerian cowpea accessions and their relationship with grain yield. Cont. J. Food Sci. Technol..

[173] Barrier-Giullot B., Casado P., Maupetit P., Jondreville C., Gatel F. (1996). Wheat phosphorus availability: 2-*in vitro* study in broilers and pigs; relationship with endogenous phytasic acitivity and phytic phosphorus content in wheat. J. Sci. Food Agric..

[174] Panzeri D., Cassani E., Doria E., Tagliabue E., Forti L., Campion B., Bollini R., Brearley C.A., Pilu R., Nielsen E. (2011). A defective abc transporter of the MRP family, responsible for the bean *lpa1* mutation, affects the regulation of the phytic acid pathway, reduces seed *myo*-inositol and alters aba sensitivity. New Phytol..

[175] Raboy V. (2007). The abcs of low-phytate crops. Nat. Biotechnol..

[176] Petry N., Egli I., Campion B., Nielsen E., Hurrell R. (2013). Genetic reduction of phytate in common bean (*Phaseolus vulgaris* L.) seeds increases iron absorption in young women. J. Nutr..

[177] Kristensen M.B., Hels O., Morberg C., Marving J., Bugel S., Tetens I. (2005). Pork meat increases iron absorption from a 5-day fully controlled diet when compared to a vegetarian diet with similar vitamin C and phytic acid content. Br. J. Nutr..

[178] Bjornrasmussen E., Hallberg L. (1979). Effect of animal proteins on the absorption of food iron in man. Nutr. Metab..

[179] Boech S.B., Hansen M., Bukhave K., Jensen M., Sorensen S.S., Kristensen L., Purslow P.P., Skibsted L.H., Sandstrom B. (2003). Nonheme-iron absorption from a phytate-rich meal is increased by the addition of small amounts of pork meat. Am. J. Clin. Nutr..

[180] Layrisse M., Martineztorres C., Leets I., Taylor P., Ramirez J. (1984). Effect of histidine, cysteine, glutathione or beef on iron-absorption in humans. J. Nutr..

[181] Taylor P.G., Martineztorres C., Romano E.L., Layrisse M. (1986). The effect of cysteine-containing peptides released during meat digestion on iron-absorption in humans. Am. J. Clin. Nutr..

[182] Martinez-Torres C., Romano E., Layrisse M. (1981). Effect of cysteine on iron-absorption in man. Am. J. Clin. Nutr..

[183] Layrisse M., Martinez C., Roche M. (1968). Effect of interaction of various foods on iron absorption. Am. J. Clin. Nutr..

[184] Storcksdieck S., Bonsmann G., Hurrell R.F. (2007). Iron-binding properties, amino acid composition, and structure of muscle tissue peptides from *in vitro* digestion of different meat sources. J. Food Sci..

[185] Laparra J.M., Tako E., Glahn R.P., Miller D.D. (2008). Isolated glycosaminoglycans from cooked haddock enhance nonheme iron uptake by caco-2 cells. J. Agric. Food Chem..

[186] Laparra J.M., Barbera R., Alegria A., Glahn R.P., Miller D.D. (2009). Purified glycosaminoglycans from cooked haddock may enhance fe uptake via endocytosis in a caco-2 cell culture model. J. Food Sci..

[187] Bonsmann S.S.G., Walczyk T., Renggli S., Hurrell R.F. (2007). Nonheme iron absorption in young women is not influenced by purified sulfated and unsulfated glycosaminoglycans. J. Nutr..

[188] Armah C.N., Sharp P., Mellon F.A., Pariagh S., Lund E.K., Dainty J.R., Teucher B., Fairweather-Tait S.J. (2008). l-α-glycerophosphocholine contributes to meat's enhancement of nonheme iron absorption. J. Nutr..

[189] Cook J.D., Monsen E.R. (1976). Food iron-absorption in human subjects 0.3. Comparison of effect of animal proteins on nonheme iron-absorption. Am. J. Clin. Nutr..

[190] Monsen E.R., Cook J.D. (1979). Food iron-absorption in human-subjects .5. Effects of the major dietary constituents of a semi-synthetic meal. Am. J. Clin. Nutr..

[191] Hurrell R.F., Lynch S.R., Trinidad T.P., Dassenko S.A., Cook J.D. (1989). Iron-absorption in humans as influenced by bovine-milk proteins. Am. J. Clin. Nutr..

[192] Cook J.D., Morck T.A., Lynch S.R. (1981). The inhibitory effect of soy products on non-heme iron-absorption in man. Am. J. Clin. Nutr..

[193] Lynch S.R., Dassenko S.A., Cook J.D., Juillerat M.A., Hurrell R.F. (1994). Inhibitory effect of a soybean-protein related moiety on iron-absorption in humans. Am. J. Clin. Nutr..

[194] Davidsson L., Dimitriou T., Walczyk T., Hurrell R. (2001). Iron absorption from experimental infant formulas based on pea (*Pisum sativum*)-protein isolate: The effect of phytic acid and ascorbic acid. Br. J. Nutr..

[195] Davidsson L., Galan P., Kastenmayer P., Cherouvrier F., Juillerat M.A., Hercberg S., Hurrell R.F. (1994). Iron bioavailability studied in infants-the influence of phytic acid and ascorbic-acid in infant formulas based on soy isolate. Pediatr. Res..

[196] Hisayasu S., Orimo H., Migita S., Ikeda Y., Satoh K., Shinjo S., Hirai Y., Yoshino Y. (1992). Soybean protein isolate and soybean lectin inhibit iron-absorption in rats. J. Nutr..

[197] Evans R.J., Bandemer S.L. (1967). Nutritive value of legume seed proteins. J. Agric. Food Chem..

[198] Mundi S., Aluko R.E. (2012). Physicochemical and functional properties of kidney bean albumin and globulin protein fractions. Food Res. Int..

[199] Montoya C.A., Lalles J.P., Beebe S., Leterme P. (2010). Phaseolin diversity as a possible strategy to improve the nutritional value of common beans (*Phaseolus vulgaris*). Food Res. Int..

[200] Carrasco-Castilla J., Hernandez-Alvarez A.J., Jimenez-Martinez C., Jacinto-Hernandez C., Alaiz M., Giron-Calle J., Vioque J., Davila-Ortiz G. (2012). Antioxidant and metal chelating activities of peptide fractions from phaseolin and bean protein hydrolysates. Food Chem..

[201] Carrasco-Castilla J., Hernandez-Alvarez A.J., Jimenez-Martinez C., Jacinto-Hernandez C., Alaiz M., Giron-Calle J., Vioque J., Davila-Ortiz G. (2012). Antioxidant and metal chelating activities of *Phaseolus vulgaris* L. Var. Jamapa protein isolates, phaseolin and lectin hydrolysates. Food Chem..

[202] Brown E., Hopper J. (1962). Red cell, plasma, and blood volume in the healthy women measured by radiochromium cell-labeling and hematocrit. Clin. Invest..

[203] Consaul J.R., Lee K. (1983). Extrinsic tagging in iron bioavailability research: A critical-review. J. Agric. Food Chem..

[204] Björn-Rasmussen E., Hallberg L., Walker R.B. (1973). Food iron-absorption in man 0.2. Isotopic-exchange of iron between labeled foods and between a food and an iron salt. Am. J. Clin. Nutr..

[205] Layrisse M., Martineztorres C., Renzi M., Velez F., Gonzalez M. (1976). Sugar as a vehicle for iron fortification. Am. J. Clin. Nutr..

[206] Cook J.D., Dassenko S.A., Lynch S.R. (1991). Assessment of the role of nonheme-iron availability in iron balance. Am. J. Clin. Nutr..

[207] Tidehag P., Hallmans G., Wing K., Sjostrom R., Agren G., Lundin E., Zhang J.X. (1996). A comparison of iron absorption from single meals and daily diets using radiofe (Fe-55, Fe-59). Br. J. Nutr.

[208] Lynch S.R., Beard J.L., Dassenko S.A., Cook J.D. (1984). Iron absorption from legumes in humans. Am. J. Clin. Nutr..

[209] Beiseigel J.M., Hunt J.R., Glahn R.P., Welch R.M., Menkir A., Maziya-Dixon B.B. (2007). Iron bioavailability from maize and beans: A comparison of human measurements with caco-2 cell and algorithm predictions. Am. J. Clin. Nutr..

[210] FAO/WHO (2004). Vitamin and Mineral Requirements in Human Nutrition.

[211] Hallberg L., Hulthen L., Garby L. (1998). Iron stores in man in relation to diet and iron requirements. Eur. J. Clin. Nutr..

[212] De Moura F., Palmer A., Finkelstein J., Haas J., Murray-Kolb L., Wenger M.J., Birol E., Boy E., Peña-Rosas J. (2014). Are biofortified staple food crops improving vitamin A and iron status in women and children? New evidence from efficacy trials. Adv. Nutr..

